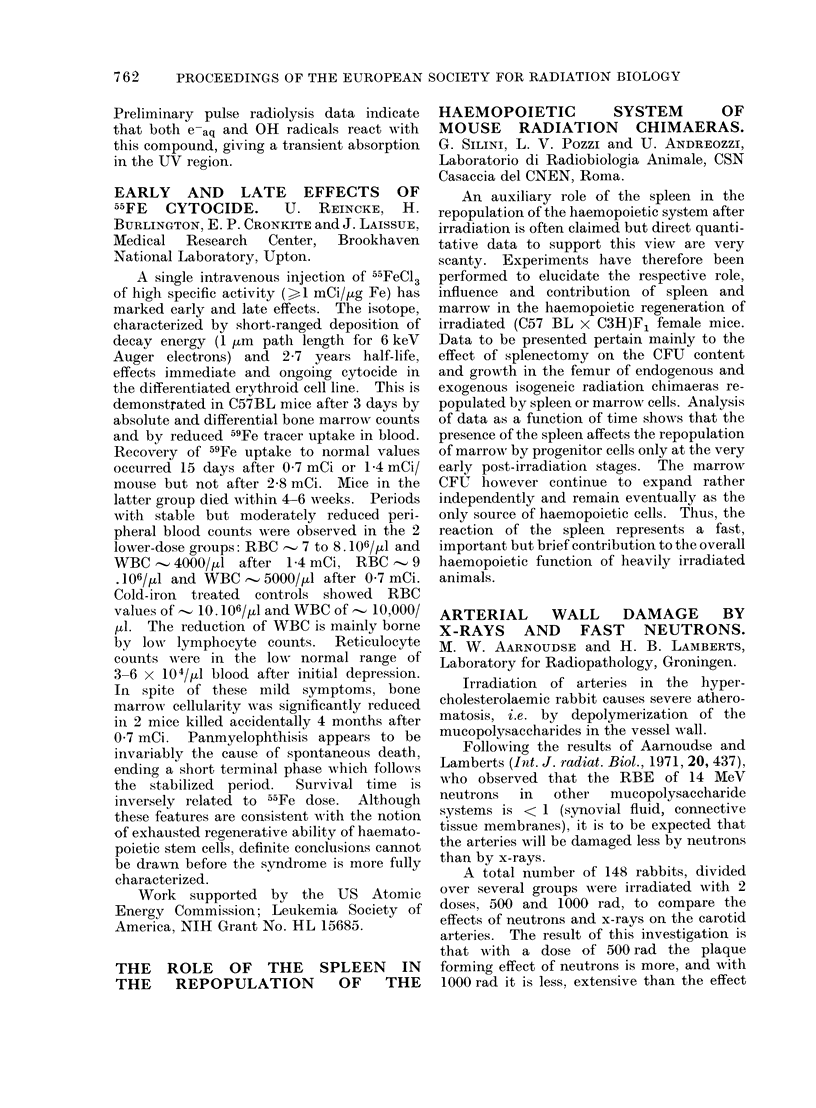# Proceedings: The role of the spleen in the repopulation of the haemopoietic system of mouse radiation chimaeras.

**DOI:** 10.1038/bjc.1975.326

**Published:** 1975-12

**Authors:** G. Silini, L. V. Pzzie, U. Andreozzi


### THE ROLE OF THE SPLEEN IN THE REPOPULATION OF THE HAEMOPOIETIC SYSTEM OF MOUSE RADIATION CHIMAERAS.

G. Silini, L. V. Pozzi and U. Andreozzi, Laboratorio di Radiobiologia Animale, CSN Casaccia del CNEN, Roma.

An auxiliary role of the spleen in the repopulation of the haemopoietic system after irradiation is often claimed but direct quantitative data to support this view are very scanty. Experiments have therefore been performed to elucidate the respective role, influence and contribution of spleen and marrow in the haemopoietic regeneration of irradiated (C57 BL $\times$ C3H)$F_1$ female mice. Data to be presented pertain mainly to the effect of splenectomy on the CFU content and growth in the femur of endogenous and exogenous isogeneic radiation chimaeras repopulated by spleen or marrow cells. Analysis of data as a function of time shows that the presence of the spleen affects the repopulation of marrow by progenitor cells only at the very early post-irradiation stages. The marrow CFU however continue to expand rather independently and remain eventually as the only source of haemopoietic cells. Thus, the reaction of the spleen represents a fast, important but brief contribution to the overall haemopoietic function of heavily irradiated animals.